# Correlation between monocyte to high-density lipoprotein ratio and major adverse cardiovascular events in patients with acute coronary syndrome after percutaneous coronary intervention

**DOI:** 10.12669/pjms.37.3.3469

**Published:** 2021

**Authors:** Rong Yu, Ruigang Hou, Tong Wang, Tianliang Li, Huiyuan Han, Jian An

**Affiliations:** 1 Rong Yu, Department of Medicine, Shanxi Cardiovascular Hospital, Taiyuan 030024, China; 2 Ruigang Hou, The Second Hospital of Shanxi Medical University, Taiyuan 030001, China; 3 Tong Wang, Department of Health Statistics, School of Public Health, Shanxi Medical University, Taiyuan 030001, China; 4 Tianliang Li, Shanxi Cardiovascular Hospital, Taiyuan 030024, China; 5 Huiyuan Han, Department of Cardiology, Shanxi Cardiovascular Hospital, Taiyuan 030024, China; 6 Jian An, Department of Cardiology, Shanxi Cardiovascular Hospital, Taiyuan 030024, China

**Keywords:** Acute coronary syndrome, High-density lipoprotein, Major adverse cardiovascular event, Monocyte, Percutaneous coronary intervention

## Abstract

**Objective:**

To investigate the correlation between monocyte to high-density lipoprotein ratio (MHR) and major adverse cardiovascular events (MACE) in patients with acute coronary syndrome (ACS) after percutaneous coronary intervention (PCI).

**Methods:**

In this retrospective study, 120 ACS patients who received PCI in our hospital from September 2014 to August 2019 were selected and divided into MACE group and normal discharge (ND) group. Their clinical data were collected, and MHR values were compared. Logistic regression analysis was conducted to analyze the correlations between various factors and ACS. The correlation between MHR and Gensini score was subjected to Pearson’s analysis. Receiver operating characteristic (ROC) curve was plotted to analyze the diagnostic value of MHR for MACE.

**Results:**

Hypertension degree, white cell count, Gensini score, MHR and the levels of total cholesterol (TC), triglyceride (TG), low-density lipoprotein cholesterol (LDLC), high-density lipoprotein cholesterol (HDLC), apolipoprotein A1 (ApoA1), ApoB, lipoprotein (a) [LP(a)] and uric acid (UA) in MACE group were significantly higher than those in ND group (P<0.05). HDLC, ApoA1, TC, MHR, LDLC and ApoB were independent risk factors for MACE of ACS patients after PCI (P<0.05). There was a positive correlation between MHR and Gensini score (r=0.832, P<0.05), and the optimal cutoff value of MHR for diagnosing MACE was 9.45.

**Conclusion:**

Serum MHR is positively correlated with Gensini score in ACS patients after PCI, which can be used as an independent predictor for MACE in hospital.

## INTRODUCTION

Acute coronary syndrome (ACS) is characterized by acute onset, poor prognosis and high mortality rate, which seriously jeopardizes the life and health of patients.^1^ The erosion or rupture of unstable plaques in the coronary artery leads to thrombosis and decrease or even interruption of myocardial blood flow, thus endangering life.^2^ Percutaneous coronary intervention (PCI) is a commonly used clinical method to control the deterioration of ACS, but not all ACS patients can benefit from it. Oxidative stress and inflammation have been proven to play vital roles in the occurrence and development of ACS.^3^ Inflammatory reaction markers, such as C-reactive protein, neutrophil-to-lymphocyte ratio and platelet-to-lymphocytes ratio, have become independent predictors for coronary artery disease and are closely associated with the prognosis of ACS. The monocyte to high-density lipoprotein ratio (MHR), as a new indicator for inflammation, is of great significance in coronary artery-related diseases.^4^ The role of MHR in ACS after PCI has never been reported. This study aimed to explore the correlation between MHR and major adverse cardiovascular events (MACE) after PCI, and to provide new predictors for high-risk ACS patients in clinical practice.

## METHODS

In this retrospective study, 120 ACS patients who received PCI in our hospital from September 2014 to August 2019 were selected, and their clinical data were collected. There were 68 males and 52 females aged 43-92 years old, with an average age of (62.04 ± 10.87) years old. All patients were divided into MACE group (n=39), including 10 cases of sudden cardiac arrest, eight cases of cardiogenic shock, 16 cases of malignant arrhythmia and five deaths, and normal discharge (ND) group (n=81). This study was approved by the Ethics Committee of the hospital (approval No. 2014037; date: August 27th, 2014), and all patients signed the informed consent.

### Inclusion criteria:

The patients were diagnosed according to the diagnostic criteria of ACS in Internal Medicine (8th edition) published by People’s Medical Publishing House Co., Ltd.:

1. Dynamically changing electrocardiograms.
2. Appearance of myocardial ischemia.
3. Lumen stenosis ≥50% in at least one main vessel (such as right coronary artery, left main artery, left circumflex branch and left anterior descending branch) shown in coronary angiography.
4. Weakened function of the ventricular wall shown in cardiac ultrasound.
5. Increased troponins.
6. Abnormal periodic movement.

### Exclusion criteria:

1. Patients with myocarditis.
2. Those who received thrombolytic therapy for coronary heart disease.
3. Those with past history of cardiac surgery.
4. Those with autoimmune diseases.
5. Those with active infection and chronic inflammatory diseases.
6. Those with liver and kidney failure or those complicated with serious medical diseases.
7. Those suffering from malignant tumors.
8. Those who were not suitable for PCI.
9. Those with incomplete clinical data.

### Criteria for performing PCI:

According to the related standards of 2010 American Heart Association Guidelines for PCI,^5^ ACS patients with PCI indications were treated with PCI.

### Gensini scoring criteria for coronary artery disease:

Clinically, coronary angiography is applied to quantitatively evaluate the stenosis degree of coronary artery disease.^6^ The product of the stenosis score of each coronary artery and the coronary segment score is the Gensini score of the coronary artery disease. The scoring criteria of coronary angiography results are shown in Table-I.

**Table I:** Gensini scoring criteria of coronary angiography results

		Score (point)
Stenosis degree	1%-25%	1
	26%-50%	2
	51%-75%	4
	76%-90%	8
	91%-99%	16
	100%	32
Lesion site	Left main artery	5
	Proximal segment of left anterior descending branch or circumflex artery	2.5
	Middle segment of left anterior descending branch	1.5
	Distal segment of left anterior descending branch	1
	Middle and distal segments of left circumflex branch	1
	Proximal, middle and distal segments of right coronary artery	1
	Small branch	0.5

### Definition of MACE:

MACE occurred during hospitalization after PCI, including cardiac death, ventricular fibrillation, congestive heart failure, malignant arrhythmia, cardiovascular atherosclerosis, cardiac arrest, hypoxic encephalopathy and ischemic stroke.

### Collection of baseline clinical data:

The baseline clinical data of patients were collected, including diabetes, hypertension, smoking history and other clinical parameters. After admission and fasting for 10 hour, 5 mL of venous blood was drawn in the early morning on the next day. The whole blood anticoagulated with dipotassium ethylenediaminetetraacetic acid (EDTA-K2) was detected using a BC-5300 full-automatic hematology analyzer, and the white blood cell count and monocyte count (M) were obtained. Then low-density lipoprotein cholesterol (LDLC), high-density lipoprotein cholesterol (HDLC), triglyceride (TG), apolipoprotein A1 (ApoA1), ApoB, lipoprotein (a) [LP(a)] and uric acid (UA) were examined using an AU5800 full-automatic chemistry analyzer (Beckman Coulter). The ratio of M to HDL was MHR.

### Coronary angiography:

The Allen test results of the included patients were negative. Following local anesthesia, the contrast agent was injected through radial artery puncture, and Siemens Artis Zeego III digital angiography machine was utilized to quantitatively assess the coronary artery stenosis. The examination was performed and the results were recorded by two experienced interventional cardiologists in our hospital.

### Statistical analysis:

All data were statistically analyzed by SPSS 20.0 software. The quantitative data conforming to normal distribution were expressed as mean ± standard deviation, and intergroup comparisons were carried out by the independent samples t test. The numerical data were represented as percentage (%), and intergroup comparisons were performed with the χ^2^ test. Logistic regression analysis was conducted to analyze the correlations between various factors and ACS. The correlation between MHR and Gensini score was subjected to Pearson’s analysis. Receiver operating characteristic (ROC) curve was plotted to analyze the diagnostic value of MHR for MACE. P<0.05 was considered statistically significant.

## RESULTS

### Baseline clinical data:

**Fig.1:**
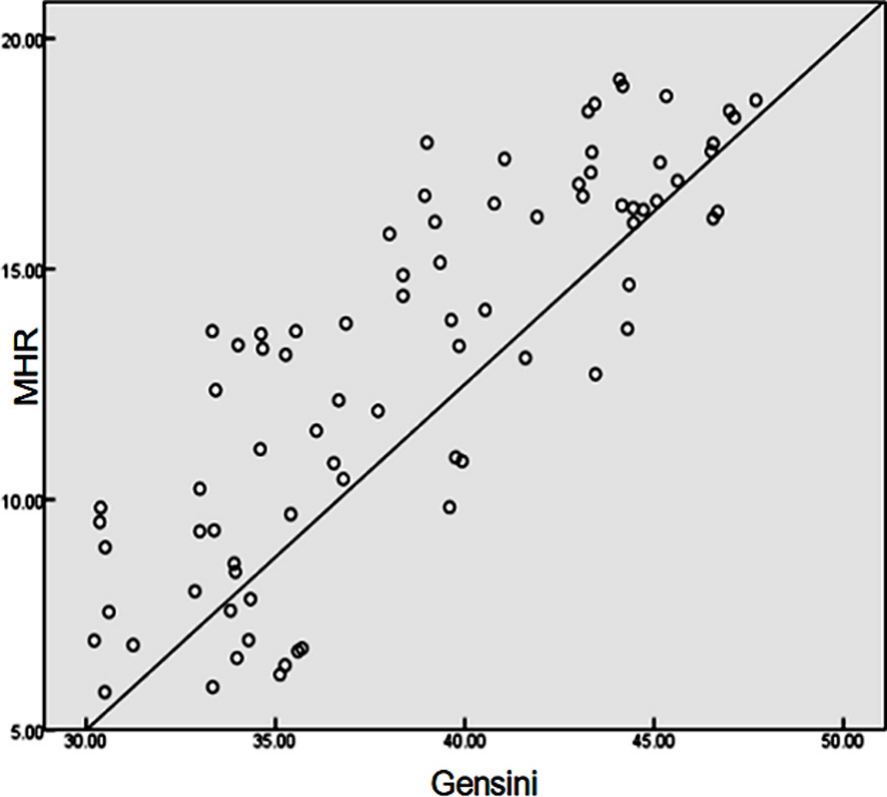
Correlation between MHR and Gensini score.

The baseline data of the two groups of patients were analyzed, and it was found that hypertension degree, white blood cell count, Gensini score, MHR and the levels of TC, TG, LDLC, HDLC, ApoA1, ApoB, LP(a) and UA in MACE group were significantly higher than those in ND group, and the differences were statistically significant (P<0.05). The remaining data such as gender, age, smoking history, diabetes, coronary heart disease, body mass index and M in the two groups were not significantly different, without statistical significance (P>0.05) (Table-II).Logistic regression analysis revealed that HDLC, ApoA1, TC, MHR, LDLC and ApoB were independent risk factors for MACE of ACS patients after PCI (P<0.05) (Table-III). The correlation between MHR and Gensini score was analyzed by Pearson’s analysis. MHR had a positive correlation with Gensini score (r=0.832, P<0.05) (Fig.1).

**Table II:** Baseline clinical data.

	MACE group (n=39)	ND group (n=81)	***P***
Male [case, (%)]	26 (66.67)	42 (51.85)	0.125
Age (year)	68.65±6.56	62.58±7.12	0.021
Smoking [case, (%)]	18 (46.15)	41 (50.61)	0.740
Hypertension [case, (%)]	27 (71.01)	39 (48.14)	0.030*
Diabetes mellitus [case, (%)]	22 (56.41)	43 (53.09)	0.730
Coronary artery disease [case, (%)]	20 (51.28)	37 (45.68)	0.560
BMI (kg/m^2^)	23.41±3.55	25.36±2.23	0.686
WBC count (×10^6^/L)	12.35±3.41	9.25±2.68	0.001*
M (×10^6^/L)	0.57±0.25	0.48±0.13	0.680
TC (mmol/L)	4.55±0.88	2.35±0.37	0.001*
TG (mmol/L)	2.69±0.29	1.60±0.35	0.006*
LDLC (mmol/L)	2.43±0.25	2.49±0.24	<0.001*
HDLC (mmol/L)	2.25±0.33	1.14±0.36	<0.001*
ApoA1 (mmol/L)	17.18±0.37	6.85±0.53	<0.001*
ApoB (mmol/L)	1.13±0.25	0.75±0.06	<0.001*
LP(a) (mmol/L)	310.36±150.52	113.96±93.25	<0.001*
UA (mmol/L)	4.56±0.88	1.25±0.29	<0.001*
Gensini score	45.87±3.85	32.57±2.71	0.03*
MHR	16.59±2.61	8.41±2.64	<0.001*

**Table III:** Logistic regression analysis results.

Index	β	SE	Wald	*P*	OR	95%CI
M	-4.03	2.98	2.62	0.15	0.08	0.01~2.72
ApoA1	-1.21	0.75	1.93	0.02	1.34	1.01~2.97
ApoB	2.34	1.08	4.26	0.01	1.12	1.07~1.39
HDLC	-1.87	0.93	3.89	0.02	1.49	1.25~3.85
LDLC	1.32	0.32	18.94	0.00	4.36	2.53~8.67
MHR	10.87	2.97	11.42	0.01	9.01	5.98~14.62
TC	-0.03	0.02	0.001	0.02	1.99	1.45~4.82
TG	0.95	0.38	8.06	0.18	2.84	0.87~5.91

### ROC curve analysis of diagnostic value of MHR for MACE:

**Fig.2:**
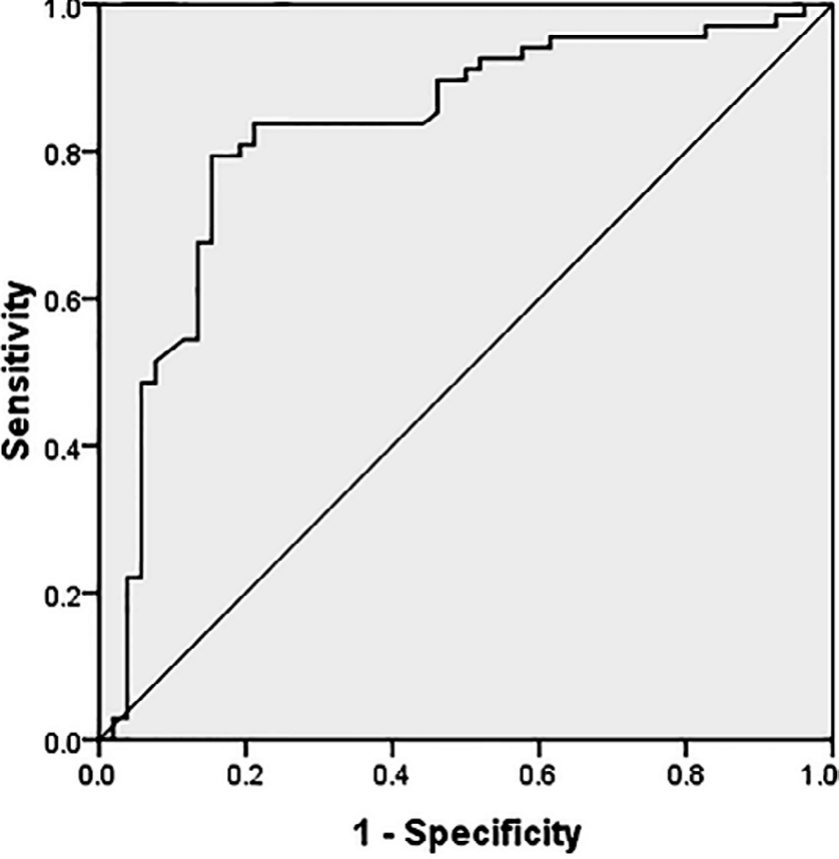
ROC curve analysis of diagnostic value of MHR for MACE.

The ROC curve of MHR was assessed, and the area under the curve was 0.827, indicating that MHR has high diagnostic value for MACE. Besides, the optimal cutoff value for diagnosing based on MHR was 9.45 (Fig.2).

## DISCUSSION

Inflammation is the key factor in the course of coronary artery disease, and persistent inflammation is the main pathological process of ACS.^7^ The abnormal expression of inflammatory factors is related to the formation, development and rupture of plaque in the course of coronary atherosclerosis.^8^ Currently, numerous inflammatory mediators or markers have become predictors for the development of coronary artery disease. In recent years, the association between MHR and the progression of ACS has attracted wide concern and is expected to become a new predictor for the course of ACS.

Abnormal blood lipid in the body exerts a very crucial effect in the deterioration of cardiovascular diseases. At present, primary hospitals in China can detect four blood lipid indices, i.e. TC, TG, LDLC and HDLC. It has been shown in a study that the change of blood viscosity triggered by lipid metabolism disorder greatly increases the risk of MACE and even death.^9^ Herein, the levels of TC, TG, LDLC and HDLC in patients in MACE group were significantly higher than those in ND group. The lipid metabolism disorder in ACS patients is triggered by the joint action of multiple effector cells and cytokines.^10^ In addition, macrophages transformed from monocytes in serum are the initiating cells of plaque formation in ACS.^11^ Macrophages phagocytize LDLC and its apolipoprotein ApoB lipid particles and deposits on the vascular endothelium.^12^ After gathering to a certain extent, macrophages transform into foam cells, and then rupture and die, thereby contributing to the malignant development of ACS. In the meantime, monocytes recruited by inflammatory stress can also facilitate the transformation of oxidized LDL and other intimal lipids into foam cells in the process of ACS plaque formation, which are continuously deposited on the intimal of blood vessels, eventually leading to coronary artery stenosis and MACE.^13^ Karabacak et al. reported that HDLC and its apolipoprotein ApoA1 promoted the efflux of TC, inhibited the activation of antigen CD11b on the activation of monocyte surface, and performed functions of anti-inflammation, anti-oxidation, anti-atherosclerosis and anti-plaque formation in the development of ACS.^14^ Therefore, MHR can be used as an inflammatory indicator to manifest coronary artery lesions, and it can reflect the lesion degree of coronary artery plaques in ACS patients from “formation mechanism” and “protection mechanism”.

Akboga et al. indicated that MHR was an independent predictor for unstable atherosclerotic plaques in patients with coronary heart disease.^15^ Cetin et al. found that MHR exhibited a positive association with ACS and showed an obvious relationship with MACEs and the clinical endpoint of ACS patients.^16^ Besides, Canpolat et al. confirmed that MHR was an independent predictor for the early relapse of paroxysmal atrial fibrillation after radiofrequency ablation.^17^ Moreover, Kundi et al. pointed out that MHR was superior to single M or HDLC concentration in predicting the occurrence and development of coronary artery lesions and cardiovascular events.^18^ In this study, the results displayed that hypertension degree, white blood cell count, Gensini score and MHR in MACE group were significantly higher than those in ND group (P<0.05). It was discovered from the logistic regression analysis that HDLC, ApoA1, TC, MHR, LDLC and ApoB were independent risk factors for MACE of ACS patients after PCI. Additionally, Pearson’s correlation analysis revealed that MHR was positively correlated with Gensini score (r=0.832, P<0.05). Lastly, ROC curve analysis proved that MHR had high diagnostic value for MACE.

### Limitations of the study:

This is a single-center study with a low number of included cases. Further multicenter prospective studies are ongoing in our group to verify the predictive value of MHR for MACE of ACS patients after PCI.

## CONCLUSION

MHR is positively correlated with Gensini score in ACS patients after PCI, and it can be used as an independent predictor for MACE in hospital. Clinicians should comprehensively analyze the clinical risks of MHR in light of the specific conditions of each patient, so as to make a more scientific and effective treatment regimen.

### Authors’ contributions:

**RY & JA:** Study design and significant manuscript revision.

**RH, TW, TL & HH:** manuscript drafting, clinical data collection and analysis.

**RY, RH, TW, TL, HH & JA:** approval of manuscript submission.

All authors are responsible and accountable for the accuracy or integrity of the work.
